# The prevalence of malaria in people living with HIV in Yaounde, Cameroon

**DOI:** 10.1186/s12889-016-3647-z

**Published:** 2016-09-13

**Authors:** Anna Longdoh Njunda, Charles Njumkeng, Shey Dickson Nsagha, Jules Clement Nguedia Assob, Tebit Emmanuel Kwenti

**Affiliations:** 1 Department of Medical Laboratory Sciences, University of Buea, P.O. Box 63, Buea, Cameroon; 2 Department of Public Health and Hygiene, University of Buea, P.O. Box 63, Buea, Cameroon

**Keywords:** Malaria, People living with HIV, Prevalence, Coinfection, CD_4_^+^ T cell count, Cotrimoxazole, ITNs, Cameroon

## Abstract

**Background:**

Coinfection with malaria and HIV is common in Sub-Saharan Africa. In the advent of a decline in the global incidence of malaria, it is important to generate updated data on the burden of malaria in people living with HIV (PLWHIV). This study was designed to determine the prevalence of malaria in PLWHIV in Yaounde, Cameroon, as well determine the association between CD_4_^+^ T cell count and malaria in the study population.

**Methods:**

In a cross sectional study performed between April 2015 and June 2016, 355 PLWHIV were enrolled and blood samples were collected for analysis. Complete blood count was performed using an automated haematology analyser (Mindray®, BC-2800) and CD_4_^+^ T cell count was performed using a flow cytometer (BD FASCount™). Giemsa-stained blood films were examined to detect malaria parasite. The Pearson’s chi-square, student’s *T*-test, ANOVA, and correlation analysis were all performed as part of the statistical analyses.

**Results:**

The prevalence of malaria observed in the study was 7.3 % (95 % CI: 4.8–10.6). No significant association was observed between the prevalence of malaria and age or gender. The prevalence of malaria was higher in participants who were not sleeping in insecticide treated bed nets, ITNs (*p* < 0.001); and in participants who were not on cotrimoxazole prophylaxis (*p* = 0.002). The prevalence of malaria (*p* < 0.001) and malaria parasite density (*p* = 0.005) were observed to be progressively higher in participants with CD_4_^+^ T cell count below 200cells/μl. Furthermore, the mean CD_4_^+^ T cell count was observed to be lower in participants coinfected with malaria compared to non-coinfected participants (323.5 vs 517.7) (*p* < 0.001). In this study, a negative correlation was observed between malaria parasite density and CD_4_^+^ T cell count (*p* = 0.019).

**Conclusions:**

A low prevalence of malaria was observed in the study population. Some of the factors accounting for the low prevalence of malaria in this study population may include the health seeking habit of PLWHIV, the use of cotrimoxazole based chemoprophylaxis, and their cautious use of ITNs.

## Background

Although the number of cases and death attributed to malaria have witnessed a decline recently, the disease is still responsible for a significant morbidity and mortality especially in sub-Saharan Africa (SSA) and in children below 5 years [1]. In 2015, there were 214 million cases of malaria worldwide, down from the 262 million cases reported in 2000, and within the same period, the number of deaths had decreased by 48 % [1]. Malaria is caused by parasitic protozoan of the genus *Plasmodium*. There are five species of *Plasmodium* which cause disease in humans namely; *Plasmodium ovale*, *P. vivax*, *P. malariae*, *P. knowlesi* and *P. falciparum*, with the latter being the most virulent accounting for the majority of cases and deaths attributed to malaria. As in most parts of SSA, *P. falciparum* is the predominant *Plasmodium spp*. in Cameroon, accounting for almost 100 % of all malaria-related cases [2].

SSA is also the region most affected by the HIV pandemic. In 2014, there were 25.8 million people living with HIV in SSA [3], accounting for approximately 70 % of the global total. Cameroon has one of the highest prevalence of HIV in West and Central Africa. In Cameroon, there were about 600,000 people living with HIV in 2013 [4]. HIV in Cameroon affects typically the poor and less privileged [5]. Because HIV is generally common among the youths, it therefore exerts a negative impact on the country’s economy (by weakening the major workforce of the country). The burden of HIV in Cameroon is also felt in the number of children who are orphaned by the disease [4, 6].

Malaria and HIV are both common in SSA because all the factors which favour transmission are present including poverty. Due to their overlapping distribution, coinfection with malaria and HIV are therefore bound to be common in the area. Coinfection with malaria and HIV is thought to have a synergistic effect, with studies reporting that repeated infection with malaria leads to a more rapid decline in CD_4_^+^ T cells overtime, meanwhile malaria coinfection with HIV results in more episodes of symptomatic malaria [7], and more episodes of severe or complicated malaria including death in both children and adults [8–12]. The risk of severe anaemia is also higher in HIV patients coinfected with malaria compared to HIV patients without malaria [13, 14].

Although the prevalence of coinfection between malaria and HIV had earlier been reported in some parts of Cameroon [12, 15], there is the need for continuous monitoring and epidemiologic enquiry to accommodate the current distribution of malaria which does not seem to be influenced only by the geographical setting but also changes overtime with variations in climatic conditions. This study was therefore designed to determine the prevalence of malaria in people living with HIV (PLWHIV) in Yaounde, Cameroon, and also to determine the association between the CD_4_^+^ T cell count and malaria. This will generate updated baseline data for clinico-epidemiologic purposes which will improve upon the management and control of both diseases.

## Methods

### Study area

This study was performed in Yaounde. Yaounde (3°52′N 11°31′E) with an average elevation of 750 m, is the capital of Cameroon. With a population of roughly 2.5million, Yaounde is second only to Douala as the largest city in Cameroon [16]. Yaoundé is a very diverse city with people from different works of life and is home to most of the administrative structures in the country. The climate of Yaounde is tropical with 2 rainy (March to June, September to November) and 2 dry seasons (December to February, July–August). The temperature here averages 23.7 °C, and Yaounde receives an average annual rainfall of 1643 mm [17]. Malaria transmission in Yaounde is holoendemic and seasonal with *Anopheles gambiae* as the main vector [18]. According to hospital records, peak malaria transmission occurs at the beginning of the rainy seasons. The prevalence of malaria in the general population of Yaounde is estimated at 35 % [19].

### Study design and setting

This was a cross-sectional study performed between April 2015 and June 2016, involving PLWHIV. Participants were recruited at the Yaounde Military Hospital (YMH). The YMH is one of the main treatment facility for HIV/AIDS patients in Yaounde receiving close to a thousand HIV/AIDS patients every month, coming from all over Yaounde and it’s environ.

### Sample size estimation

Using the following formula for sample size calculation [20];

$$ \mathrm{n} = \frac{Z^2 \times p\left(1- p\right)}{e^2} $$

Z = 1.96

p = prevalence of malaria among PLWHIV = 29.4 % [12].

e = error rate = 0.05

$$ \mathrm{n} = \frac{1.96^2 \times 0.294\left(1-0.294\right)}{0.05^2} = 319 $$

It was estimated that we will require a sample of at least 319 PLHIV.

### Sampling technique

A convenient sampling technique was used, where PLHIV were consecutively recruited into the study as they came for consultation or to collect their ARV drugs.

### Study population

PLWHIV were recruited at the HIV treatment center of the Yaounde Military Hospital. The study population consisted of HIV/AIDS patients on treatment and newly diagnosed cases (treatment-naïve). To be included in the study, participants were to be male or female of all ages. Participants were not to be on any antimalarial medication one week prior to the study. Participants were also required to provide a written informed consent, which was duly explained to them in English, French or the local Pidgin English. For minors and those who could not sign the informed consent, their parents, guardians, or next of kin did on their behalf.

### Sample collection

Once participants gave their informed consent, their blood samples were collected. About 4 ml of blood was collected into EDTA anticoagulated test tubes following aseptic techniques, to perform the complete blood count (CBC) and CD_4_^+^ T cell count. Thick and thin blood films were also prepared for malaria microscopy.

### Performance of CBC and CD_4_^+^ T cell counts

The CBC was performed using the Mindray® automatic haematology analyser (BC-2800, Shenzhen Mindray Bio-Medical Electronics Co., Ltd, Shenzhen, P.R., China).

CD_4_^+^ T cell counts were determined using a flow cytometer, BD FASCount™.

### Detection of malaria parasites

Thick and thin blood films were prepared and stained with 10 % Giemsa and examined using methods previously described [21]. Blood films were read by two expert microscopists who were blinded from the results of the other. In case of any discrepancy with the results obtained by the two microscopists, a third was brought in and the results he gave was considered as final. The thick films were screened for at least 200 fields using the 100X (oil immersion) objective. If asexual parasites were observed, the density was then determined by counting the number of parasites against 500 leucocytes. The slides were only declared negative after counting to 2500WBC. The parasite density was obtained by dividing the number of parasites by 500 and multiplying the result by the actual white blood cell count of the patient obtained from the CBC results [22].

### Statistical analysis

Data collected were entered into an Excel spreadsheet and analysed using the Stata® version 12.1 software (StataCorp LP, Texas, USA). The statistical tests performed included the Pearson’s Chi-square for comparison of proportions, the Student’s *T*-test and ANOVA for the comparison of group means, and correlation analysis to determine the association between parasite density and CD_4_^+^ T cell count. Statistical significance was set at *p* < 0.05.

## Results

Four hundred and seven PLWHIV were approached, 355 of them meet the inclusion criteria and were therefore enrolled PLWHIV successfully took part into the study. Among them were 236 (66.5 %) females and 119 (33.5 %) males (Table 1). The ages of the participants ranged between 5 and 72 (mean ± SD = 35.29 ± 12.26). The mean duration of HIV was 3.0 ± 3.3 years. A majority (46.2 %) of the participants had a CD4+ T cell count above 500cells/μl. A bulk (78.9 %) of the participants reported to have been using insecticide treated bed nets (ITNs). Among the participants, 280 (78.9 %) were on antiretroviral therapy (ART) at enrollment, and 234 (65.9 %) were on prophylaxis with cotrimoxazole (Table 1).

**Table 1:** Clinical and demographic characteristics of the study population

Parameter		n (%)
Age	<20	26 (7.3)
	20–39	208 (58.6)
	40–59	107 (30.1)
	≥60	14 (3.9)
	Total	355
Gender	F	236 (66.5)
	M	119 (33.5)
	Total	355
On HAART	Yes	201 (56.6)
	No	154 (43.4)
	Total	355
Sleeping under ITN	Yes	280 (78.9)
	No	75 (21.1)
	Total	355
Mean (±SD) duration of HIV	HAART	4.4 ± 2.5
	Naïve	1.2 ± 3.4
	Total	3.0 ± 3.3
CD_4_+ T cell counts	<200	40 (11.3)
	200–499	151 (42.5)
	≥500	164 (46.2)
	Total	355
On prophylaxis with cotrimoxazole	Yes	234 (65.9)
	No	121 (34.1)
	Total	355

Twenty six (26) of the 355 PLWHIV were infected with malaria parasite giving a prevalence of 7.3 % (95 % CI: 4.8–10.6). The prevalence of malaria was higher in individuals between 40 and 59years of age (Table 2). However no significant difference was observed between the prevalence of malaria and age (*p* = 0.960). The prevalence of malaria was higher in males compared to females (Table 2). Like the age, no significant association was observed between prevalence of malaria and gender in this study (*p* = 0.388). The prevalence of malaria was significantly higher in participants that were not sleeping under ITNs compared to those who were (p <0.001). The prevalence of malaria was also significantly higher in participants who were not on prophylaxis with cotrimoxazole (*p* = 0.002) (Table 2).

**Table 2:** The distribution of malaria parasitaemia with respect to age, gender, HIV treatment status, CD_4_+ T cell counts, use of ITNs and cotrimoxazole prophylaxis

Parameter		n (%)	Malaria positive n (%)	*χ* ^2^	p-value
Age	<20	26	2 (7.7)	0.3	0.960
	20–39	208	14 (6.7)		
	40–59	107	9 (8.4)		
	≥60	14	1 (7.1)		
	Total	355	26 (7.3)		
Gender	F	236	15 (6.4)	0.324	0.388*
	M	119	11 (9.2)		
	Total	355	26		
On HAART	Yes	201	13 (6.5)	0.479	0.540*
	No	154	13 (8.4)		
	Total	355	26		
Sleeping under ITN	Yes	280	8 (2.9)	39.0	<0.001
	No	75	18 (24.0)		
	Total	355	26		
CD4+ T cell counts	<200	40	11 (27.5)	28.5	<0.001
	200–499	151	10 (6.6)		
	≥500	164	5 (3.1)		
	Total	355	26		
On prophylaxis with cotrimoxazole	Yes	234	10 (4.1)	9.7	0.002
	No	121	16 (13.2)		
	Total	355	26		

The CD_4_^+^ T cell count of the participants ranged between 38 and 1600 cells/μl (mean ± SD = 503.5 ± 276.2). The prevalence of malaria parasitaemia was higher in participants with CD_4_^+^ T cell count below 200 cells/μl and lowest in participants with counts above 500cells/μl (Table 2). A significant association was observed between prevalence of malaria and CD_4_^+^ T cell count (*p* < 0.001). Furthermore, the mean CD_4_^+^ T cell count was lower in patients coinfected with malaria parasites compared to non-coinfected participants (323.5 vs 517.7). The difference in the CD_4_+ T cell counts between malaria coinfected patients and non-coinfected patients was observed to be significant statistically (*p* < 0.001).

**Fig. 1:**
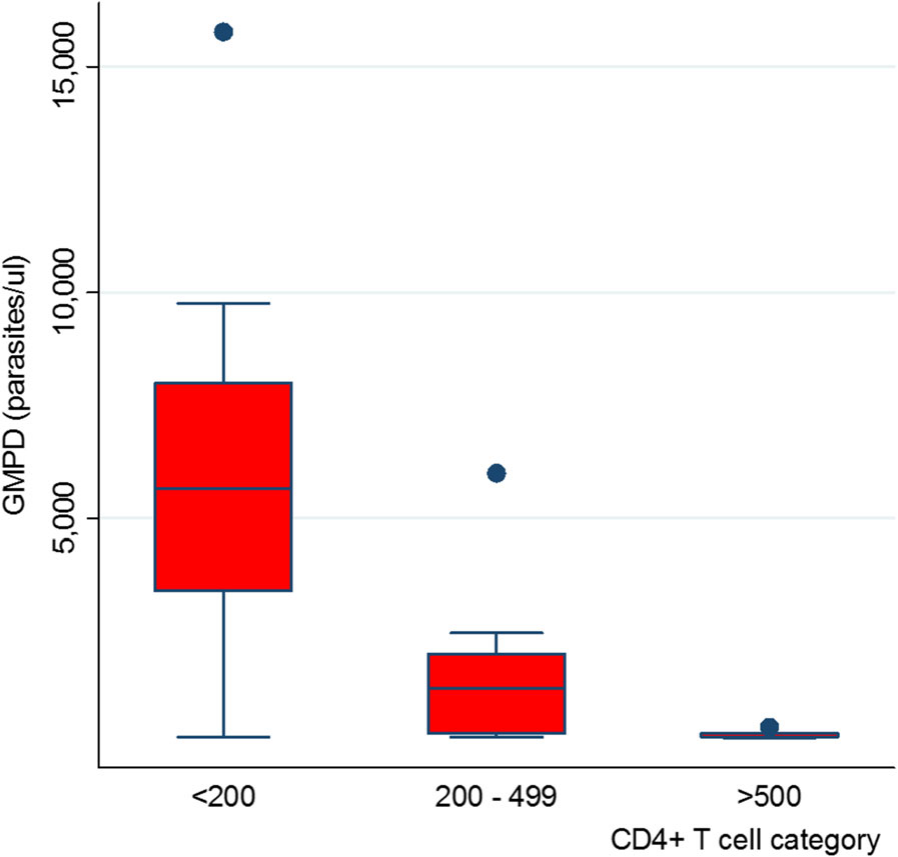
Distribution of the GMPD according to the different categories of CD_4_^+^ T cell count shows a progressively higher GMPD in patients with CD_4_^+^ T cell count below 200cells/μl (*p* = 0.005)

**Fig. 2:**
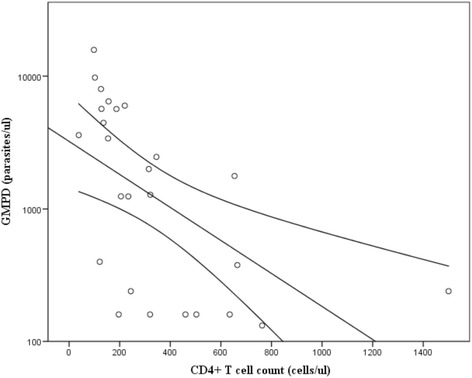
Fitted plot of CD_4_^+^ T cell count against malaria parasite density showing a negative correlation (*r* = −0.465, *p* = 0.019)

The geometric mean parasite density (GMPD) observed was 1219.7 parasites/μl. The GMPD was progressively higher in CD_4_^+^ T cell count below 200 cells/μl (Fig. 1). A Comparison between GMPD and the CD_4_^+^ T cell count category revealed a significant association (*p* = 0.005). Furthermore a negative correlation was observed between CD_4_^+^ T cell count and GMPD (*r* = −0.465, *p* = 0.019) (Fig. 2).

## Discussion

Due to the overlapping distribution of malaria and HIV, coinfection between malaria and HIV are therefore common in SSA. With the declining trend in the global incidence of malaria [1], it is important to generate updated data on the burden of malaria in HIV. This study was aimed at determining the prevalence of malaria in PLWHIV in Yaounde, as wells as investigate the association between CD_4_+ T cell count and malaria.

In the current study. A prevalence of 7.3 % for malaria parasite was observed. This prevalence is lower compared to the prevalence of 34 % for malaria reported in the general population of Yaounde [19]. This prevalence is however higher compared to the 2.24 % reported in PLWHIV in Bamenda in the Northwest region of Cameroon [15]. Furthermore, the prevalence observed in this study is lower compared to similar studies performed in other countries; 11.75 % in Ghana [14], and 18.5 % in Nigeria [23]. The difference between the prevalence observed in these studies and ours could be due to the geographical differences in the study populations and the differences in the level of malaria endemicity. Malaria in Yaounde can be described as holoendemic and seasonal [18]. The prevalence is also very low compared to the prevalence of malaria in the other high risk groups including children (where prevalence could be as high as 98 % in some settings) [24], and pregnant women [25]. The low prevalence of malaria in this group could be attributed to the health seeking attitude of HIV patients. Studies have shown that malaria in HIV is more severe [8–13] and patients infected with malaria will quickly go down with the disease and seek medical attention faster. Another factor that could be responsible for the low prevalence of malaria in the target population is the use of cotrimoxazole (CTX) based chemoprophylaxis which is recommended for the protection against opportunistic infection in all PLWHIV in Cameroon. In this study, the prevalence of malaria among participants who were not on prophylaxis with cotrimoxazole was significantly higher than those who were on cotrimoxazole prophylaxis (*p* = 0.002). Trimethoprim-Sulfamethoxazole which is the active ingredient of CTX is also effective therapeutically against malaria [26, 27]. A study performed in Mozambique had shown that patients that were on CTX had lower risk of malaria compared to those that were not on CTX [13]. Regular use of ITNs could also be responsible for the low prevalence of malaria as evident from this study that 78.9 % of the patients reported to have been cautiously sleeping in ITNs bearing in mind their current status. ITNs have been made available to almost every household in Cameroon thanks to government efforts to increase coverage and encourage usage. In this study, the prevalence of malaria was significantly (p <0.001) higher in participants that were not sleeping under ITNs compared to those who were (24 % vs. 2.9 %), which confirms the benefit of using the ITNs. This finding is contrary to the study by Njunda et al. [15] in which no association was observed between the use of ITNs and prevalence of malaria.

In the current study, no significant association was observed between the prevalence of malaria and age (*p* = 0.960) or gender (*p* = 0.388). These findings echo earlier reports by Njunda et al. [15] and Ojurongbe et al. [23]. The majority of the study participants were adults which represent a low risk group for malaria unlike children (especially those below 5 years) who represent the most at risk group [1]. This may also have accounted for the low prevalence of malaria in this study. Studies investigating the burden of malaria in HIV-infected children will be required in the study area to provide a clearer picture. In the current study, no significant difference was observed in the prevalence of malaria between PLWHIV on ART and treatment naïve group (*p* = 0.540). This finding is in resonance with the study by Kimbi et al. [28].

Overall, the mean CD_4_^+^ T cell count observed was 503.5 cells/μl. As recommended by the government, treatment of HIV/AIDS in Yaounde and Cameroon as a whole commences at diagnosis and not dependent on the CD_4_^+^ T cell count as it was before. This may explain the generally high CD_4_^+^ T cell count of the study population. The prevalence of malaria was progressively higher in patients with CD_4_^+^ T cell count below 200cells/μl (*p* < 0.001). This is contrary to the study performed by Iroezondu et al. [29]. Furthermore, the CD_4_^+^ T cell count was significantly lower in HIV patients coinfected with malaria compared to those without, which confirms the study by Tay et al. [14] in which patients with malaria were also observed to have lower CD_4_^+^ T cell count. The finding of a significant association between malaria prevalence and CD_4_^+^ T cell count is contrary to studies performed elsewhere [23, 25].

The GMPD observed in this study was 1219 parasites/μl. The GMPD was also observed to be progressively higher in patients with CD_4_^+^ T cell count below 200cells/μl (*p* = 0.005). This increasing parasite density with decreasing CD_4_^+^ T cell count could be attributed to the weakening of the immune responses of these patients. Both the cellular and humoral immune responses are thought to play a role in the protection against malaria [30], and their activity is regulated by the CD_4_^+^ T cell. A depletion in the number of CD_4_^+^ T cells will therefore affect the cellular and humoral immune responses and consequently the immune responsiveness to malaria antigens [31, 32]. As the immunity weakens, it allows for the parasites to multiply hence disease progression. The finding of a significant negative correlation in this study further supports this hypothesis.

In the current study, only participants who attended the HIV/AIDS treatment center of the Yaounde Military Hospital were recruited. This may be biased against PLWHIV do not attend the facility or who did not come to the facility within the study timeframe. This may not give a true representation of PLWHIV in Yaounde. This serves as a major limitation of the study. In addition, the study was performed during the rainy season, a period well known for high malaria transmission. This also, may have affected the true representation of the prevalence of malaria in the study population, as data collected during the dry season may be lower. Studies to determine how the prevalence of malaria among PLWHIV varies with respect to season will therefore be needed in the study area.

## Conclusion

A low prevalence of malaria was observed among PLWHIV in Yaounde, which is lower compared to prevalence reported in the general population of Yaounde. The prevalence was also lower compared to the prevalence reported in other countries in Sub-Saharan Africa. The low prevalence of malaria could be attributed to the wide use of insecticide treated bed nets and the cotrimoxazole based chemoprophylaxis in the study population. The prevalence of malaria parasitaemia was not observed to be associated with age or gender. The findings of a high prevalence of malaria and a higher parasite density in participants with CD_4_^+^ T cell count below 200cells/μl, as well as a negative correlation between the parasite density and the CD_4_^+^ T cell count is indicative of the role of the immune response in the protection against malaria in the target population.

## Abbreviations

ART: Antiretroviral therapy
CD: Cluster of differentiation
CTX: Cotrimoxazole
GMPD: Geometric mean parasite density
HIV: Human Immunodeficiency Virus
ITN: Insecticide treated bed nets
PLWHIV: People living with HIV
SSA: Sub-Saharan Africa
